# Relationship between handedness and the incidence of spinal changes in the frontal plane: evaluation using Idiag® Spinal Mouse®

**DOI:** 10.1080/07853890.2021.1896654

**Published:** 2021-09-28

**Authors:** C. Castro, J. Neves Silva, E. Matos, S. Xavier Sousa, D. Gonçalves, N. Azevedo, L. Rodrigues, G. Pacheco

**Affiliations:** ISAVE – Instituto Superior de Saúde, CICS – Centro Interdisciplinar em Ciências da Saúde, Portugal

## Abstract

**Introduction:**

In middle and highschool, where classroom furniture and equipment are developed mainly for right-handed children, left-handed children may find specific obstacles that can lead to postural changes in the future [1]. The purpose of this study is to determine whether there is an association between handedness and the incidence of spinal changes in the frontal anatomical plane, in a sample of students aged between 10 and 18 years old from public middle schools in Amares, Braga, Portugal.

**Material and methods:**

A cross-sectional study was carried out with 479 students, 246 (51.4%) females and 233 (48.6%) males, aged between 10 and 18 years old ($\bar{x}$ = 13.6 years old, *δ* = 2.496). An informed consent was signed through their educational representative, after which an individual inquiry has been given to each student, regarding their sociodemographic information, activities of their daily lives and clinical history. Measurements of weight and height were individually performed, simultaneously with a dynamic evaluation of the spine in the frontal anatomical plane using the non-invasive measuring instrument Idiag® Spinal Mouse®. All data were analysed using descriptive and inferential statistics, which were performed using the Chi-Square (*χ*^2^) test for association. Significance levels (denoted as *α*) of 0.05 and 0.01 have been considered for the presence of statistically significant association between the considered variables.

**Results:**

Of all participants in the study, 431 (89.98%) were right-handed and 48 (10.02%) left- handed. The presence of left convex scoliosis was identified in the vast majority of students, particularly in the lumbar region (478 students, 99.79%), irrespective of the handedness of the student. No statistically significant association was identified between handedness and the prevalence of spinal changes in the thoracic region (*χ*^2^ = 1.355; *p*-value = .508), lumbar region (*χ*^2^ = 0.112; *p*-value = .738) and sacral region (*χ*^2^ = 2.590, *p*-value = .274) of the frontal plane. However, 86% of the students presented thoracolumbar scoliosis in "C" with convexity to the left side and 10% presented thoracolumbar scoliosis in “S”, that is, with two curves present. The limitations of this investigation were using a small sample from only one region of Portugal, not evaluating the cervical region (instrument limitation), having a small percentage of left- handed people and little time to carry out the study.

**Discussion and Conclusion:**

Although there is no significant association between laterality and scoliosis, data supported by previous studies [1,2], we consider that an early diagnosis of postural changes prevents its progression and its future appearance. Scoliosis, due to unilateral mechanical forces, may destabilise the muscle-articular joint and favour the appearance of muscular and coordinative differences and the lack of a normal movement in one area, what will result in excessive movement in another causing mechanical overload in these structures, which are sensitive to pain. These results may encourage the development of new studies to identify the causes of scoliosis in students in this age group.
